# Supplementary material for: The effects of vaginal gel from Myrtus communis on the sexual function of married women during reproductive aging: A study protocol for a randomized controlled trial
Source: PLoS One. 2026 Jan 13;21(1):e0338732. doi: 10.1371/journal.pone.0338732 (PMC12798978; doi:10.1371/journal.pone.0338732)
Supplement: S1 Fig — (DOCX) [file pone.0338732.s001.docx]

**Figure 1.** Timeline of study visits and assessment points for key variables: informed consent, eligibility criteria, randomization, sociodemographic and reproductive health data, FSFI, DASS-21, Most Bothersome Symptom (MBS), drug accountability, and adverse event reporting.

|  | Screening | V0 (Baseline) | Follow up (week 1) | Follow up (week 2) | Follow up (week 3) | V1  (Month 1) |
| --- | --- | --- | --- | --- | --- | --- |
| Informed consent |  |  |  |  |  |  |
| In/exclusion criteria |  |  |  |  |  |  |
| Randomization |  |  |  |  |  |  |
| Sociodemographic and Reproductive Health Questionnaire |  |  |  |  |  |  |
| Female Sexual Function Index (FSFI) questionnaire |  |  |  |  |  |  |
| Depression, Anxiety, and Stress Scale (DASS-21) questionnaire |  |  |  |  |  |  |
| Most Bothersome Symptom  Scale for Vaginal Atrophy (MBS) |  |  |  |  |  |  |
| Drug account |  |  |  |  |  |  |
| Adverse event reporting |  |  |  |  |  |  |
