# Supplementary material for: The effects of vaginal gel from Myrtus communis on the sexual function of married women during reproductive aging: A study protocol for a randomized controlled trial
Source: PLoS One. 2026 Jan 13;21(1):e0338732. doi: 10.1371/journal.pone.0338732 (PMC12798978; doi:10.1371/journal.pone.0338732)
Supplement: Attachment — Submitted filename: point-by-point response to the comments.docx [file pone.0338732.s004.docx]

**Dear editor,**

**Ref: PONE-D-25-17029**

**Re: "** **The effects of vaginal gel from Myrtus communis on the sexual function of married women during reproductive aging: a study protocol for a randomized controlled trial "**

I appreciate very much the comments of editor toward the improvement of the paper and I hope that a suitable revision to addresses editor' comments is carried out. Responses to the comments along with a description of the changes made on the manuscript are given below. The amended parts have been highlighted in **YELLOW** in the manuscript.

I would be glad to respond to any further questions and comments that you may have.

Sincerely yours,

Corresponding author

Dr. Raheleh Babazadeh

**Comments from Reviewer 1**

***Comment 1:*** Please upload a copy of your study protocol that was approved by your ethics committee/IRB as a Supporting Information file. By the study protocol, we mean the complete and detailed plan for the conduct and analysis of the trial approved by the ethics committee/IRB. Please send this in the original language. If this is in a language other than English, please also provide a translation.

***Response:*** The study protocol approved by the ethics committee has been submitted as a Supporting Information file. Its English translation has also been attached and submitted.

***Comment 2:*** Please note that the Study Protocol article type is only suitable for proposals of studies that have not yet started or that are ongoing. Studies that have completed either recruitment or data collection will not be considered. Please ensure that your Methods section reports the status and timeline of the study, including an estimate of when A) participant recruitment will be completed (if applicable), B) data collection will be completed and C) results are expected. If any of these stages have already been completed, this should be indicated in the Methods section.

***Response:*** The estimated timelines for the completion of participant recruitment, data collection, and availability of results have been included in the Recruitment section of the Methods. (Page: 5, line: 150-155).

Please note that due to unforeseen technical issues encountered during the course of the study, the initiation of recruitment began later than originally estimated.

***Comment 3:*** We note that you have indicated that you received funding for your study. If you received peer reviewed external funding, your submission may be considered for expedited editorial assessment. To be considered for expedited review, please upload the Funding letter you received, and English translation, as an ‘Other’ file. Please also provide proof that the funder administered independent peer review of your protocol. Examples of evidence include: a link to a description of the funder’s peer-review process; a letter from the funding agency confirming the independent peer review process; a list of the names and credentials of the peer reviewers (if available).

***Response:*** The funding approval letter, which was granted through a peer-reviewed process at Mashhad University of Medical Sciences, along with its English translation, has been submitted as an ‘Other’ file.

***Comment 4:*** We note that you did not include a copy of the original, (signed or stamped) dated IRB/research ethics committee approval letter or waiver for all study sites as an ‘Other’ file. In keeping with our submission guidelines, please upload the approval letter or waiver as an ‘Other’ file.

***Response:*** The ethics committee approval letter has been uploaded as an ‘Other’ file.

***Comment 5:*** Please complete all items on the Human Participants Research Checklist that are relevant for your submission, by following this link

***Response:*** The requested Human Participants Research Checklist has been completed and uploaded as an ‘Other’ file.

***Comment 6:*** Please amend the Methods section of your manuscript to include the date in which the IRB or Ethics committee approved or waived your study.

***Response:*** The date of approval of the study by the Ethics Committee has been added to the manuscript. (Page: 9, line: 314)

***Comment 7:*** Please remove your figures from within your manuscript file, leaving only the individual TIFF/EPS image files. These will be automatically included in the reviewer’s PDF.

***Response:*** As per your request, the table has been removed from the manuscript file and placed only in the S1 Fig.tif file. Additionally, the location of the table within the manuscript file has been indicated. (Page: 7, line: 239).
