# Supplementary material for: The effects of vaginal gel from Myrtus communis on the sexual function of married women during reproductive aging: A study protocol for a randomized controlled trial
Source: PLoS One. 2026 Jan 13;21(1):e0338732. doi: 10.1371/journal.pone.0338732 (PMC12798978; doi:10.1371/journal.pone.0338732)
Supplement: Attachment — Submitted filename: PONE-D-25-17029.docx [file pone.0338732.s003.docx]

The following major issues need to be addressed:

1- sample size:

The sample size calculation presented in the manuscript contains several concerning issues that require immediate attention. The authors reference a study by Abbasi Pirouz et al. reporting mean differences of 24.5 ± 4.5 in the intervention group and 3.4 ± 5.8 in the control group, leading to a calculated sample size of "two participants using the formula for the mean of two independent populations." This calculation appears to be fundamentally flawed.

First, the formula presented (n = 2(z₁₋α + z₁₋β)²/f²) is incomplete and lacks proper definition of the effect size parameter 'f'. The authors then state they calculated an effect size of 0.7 and arrived at 32 participants per group, but the mathematical steps are not shown or justified. The jump from 2 participants to 32 participants per group is not explained, and the 20% dropout rate adjustment to reach 38 participants per group lacks proper justification.

The authors should provide a complete, step-by-step sample size calculation including: (1) clearly defined primary outcome and clinically meaningful difference, (2) expected standard deviation based on pilot data or literature, (3) specified Type I and Type II error rates, (4) detailed calculation showing all mathematical steps, and (5) justification for the dropout rate assumption.

2- The manuscript does not specify who will generate the randomization sequence, how allocation concealment will be maintained, or what procedures will prevent selection bias.

3- The FSFI cutoff score of <28 should be referenced to validation studies in the Iranian population

•The requirement for "at least basic literacy skills" needs operational definition

•The criterion of "sexual activity at least once a week" may be too restrictive and could introduce selection bias

•The exclusion of women with "vaginal atrophy" requires clear diagnostic criteria

4- Discuss the following: (1) informed consent procedures and content, (2) procedures for obtaining consent from participants with limited literacy, (3) confidentiality protections given the sensitive nature of sexual health research, and (4) procedures for managing participants who develop concerning symptoms during the study.

5- Lines 267-275: Statistical analysis section needs expansion to include missing data handling, interim analyses, and multiple comparison procedures.

6- Lines 185-205: Inclusion criteria should be justified or reconsidered, particularly the literacy requirement and weekly sexual activity criterion.
