# Supplementary material for: The effects of vaginal gel from Myrtus communis on the sexual function of married women during reproductive aging: A study protocol for a randomized controlled trial
Source: PLoS One. 2026 Jan 13;21(1):e0338732. doi: 10.1371/journal.pone.0338732 (PMC12798978; doi:10.1371/journal.pone.0338732)
Supplement: Attachment — Submitted filename: point by point response to reviewers.docx [file pone.0338732.s006.docx]

**Dear Editor,**

**Ref:**

**Re: "** **The effects of vaginal gel from Myrtus communis on the sexual function of married women during reproductive aging: a study protocol for a randomized controlled trial "**

I appreciate very much the comments of reviewers toward the improvement of the paper and I hope that a suitable revision to addresses reviewers' comments is carried out. Responses to the comments/questions of the reviewers along with a description of the changes made on the manuscript are given below. The amended parts have been highlighted in **YELLOW** in the manuscript.

I would be glad to respond to any further questions and comments that you may have.

Sincerely yours,

Corresponding author

Dr. Raheleh Babazadeh

**Comments from Reviewers**

***Comment 1:*** Line 147 should be written in past tense.

***Response:*** Line 147 has been changed to past tense.

***Comment 2:***  Recruitment. Lines 150-155 should be written in past tense. Similar observations were made in Sampling, Lines 212-226, Interventions and control, Lines 229-250, Blinding, Lines 269-275, Adherence Monitoring and Missing Data Management, Lines 278-283, Ethical Consideration, Lines 286-301 and Statistical Methods Lines 305-323

***Response:*** As requested, lines 150–155 have been changed to past tense. The tense of the sentences in Sampling (Lines 212–226), Interventions and Control (Lines 229–250), Blinding (Lines 269–275), Adherence Monitoring and Missing Data Management (Lines 278–283), Ethical Consideration (Lines 286–301), and Statistical Methods (Lines 305–323) has been changed to the past tense.
