# Supplementary material for: The effects of vaginal gel from Myrtus communis on the sexual function of married women during reproductive aging: A study protocol for a randomized controlled trial
Source: PLoS One. 2026 Jan 13;21(1):e0338732. doi: 10.1371/journal.pone.0338732 (PMC12798978; doi:10.1371/journal.pone.0338732)
Supplement: Attachment — Submitted filename: Reviewer Comment Vaginal gel Mythus.docx [file pone.0338732.s002.docx]

**Reviewer Comment**

This study titled “The effects of vaginal gel from Myrtus communis on the sexual function of married 6 women during reproductive aging: a study protocol for a randomized controlled trial” describes a phase II clinical trial that randomly assigns participants to receive either a Myrtus communis vaginal gel or a placebo, without anyone knowing who gets what, to see how it affects sexual function in married women who can have children**.** The manuscript is well organized and provides comprehensive methodological details, including rationale, trial registration, intervention design, and statistical plans. It addresses a clinically relevant issue, female sexual dysfunction (FSD), with a culturally appropriate, natural, and potentially low-risk intervention.

1. Provide a concise overview of previous clinical trials that utilized comparable natural agents. Briefly mention the limitations of previous herbal trials or lack of data on **M.communis’s** female sexual function.
2. The explanation of the extraction and gel preparation process is too technical for general readership. Simplify technical steps and place more emphasis on dosing rationale, safety, and placebo matching.
3. The description does not include any sensory testing or blinding validation, such as odor or consistency. Describe the methods used to confirm placebo similarity (visually, chemically, or through a pilot test).
4. There was no mention of secondary outcomes (e.g., safety, side effects, satisfaction). Consider adding secondary endpoints such as participant satisfaction or incidence of adverse effects.
5. The explanation of randomization is too detailed and may cause confusion. Please simplify the block method description and include a CONSORT-style flow diagram.
6. There is no mention of managing missing data or conducting adherence analysis. Briefly describe the intention-to-treat or per-protocol approach and any imputation plan.

Overall, the study addresses a relevant clinical and cultural issue using a natural product with a solid pharmacological basis. The protocol is thorough and reflects high-quality trial planning, especially in randomization and blinding. However, the manuscript would benefit from language refinement, a more concise introduction, clearer reporting of placebo integrity and secondary endpoints, and a plan for handling missing data. I recommend minor revisions prior to acceptance.
